# Comment on “Systematic Review and Meta-Analysis of Diagnostic Accuracy of miRNAs in Patients with Pancreatic Cancer”

**DOI:** 10.1155/2018/6904569

**Published:** 2018-10-22

**Authors:** Rama Jayaraj, Chellan Kumarasamy, Madurantakam Royam Madhav, Venkatesh Pandey, Shanthi Sabarimurugan, N. Ramesh, K. M. Gothandam, Siddhartha Baxi

**Affiliations:** ^1^College of Health and Human Sciences, Charles Darwin University, Ellengowan Drive, Darwin, 0909 Northern Territory, Australia; ^2^University of Adelaide, North Terrace Campus, Adelaide, SA 5005 South Australia, Australia; ^3^School of Biosciences and Technology, Vellore Institute of Technology (VIT), Vellore, Tamil Nadu, India; ^4^South West Radiation Oncology Service, Senior Clinical Oncologist, Genesis Cancer Care Centre, Bunbury, 6230 Western Australia, Australia

Pancreatic cancer is one of the most malignant and aggressive cancers, with poor survival rates and diagnosis being based on nonspecific tumour biomarkers. Due to this, the publication, “Systematic Review and Meta-Analysis of Diagnostic Accuracy of miRNAs in Patients with Pancreatic Cancer”, by Sun et al. has excellent potential for highlighting potential miRNAs as high-accuracy diagnostic markers in pancreatic cancer [1]. However, we believe that despite the comprehensive study conducted by Sun et al., there are a few improvements that could be made to heighten the clinical utility of the paper further. As it stands, we believe that the study has some issues that prevent such applications of Sun et al.'s study.

The authors have stated that there is a need to measure the diagnostic value of miRNA in pancreatic cancer. However, previous studies in the field already exist [2–4]. Despite the previous studies reporting inconclusive results, the authors still need to highlight the differences between this study and previous publications, as well as elaborate more on its benefits over previous such studies, other than merely stating discordant results.

Another issue is that sensitivity and specificity did not differ across all the included studies. This is applicable only in a scenario where all the studies included in the pooled meta-analysis have a singular diagnostic cut-off point [5]. However, such homogeneous standards are not available when comparing multiple individually conducted studies. This scenario is only usually possible in diagnostic estimation in laboratory conditions and is difficult to achieve and replicate in a clinical setting.

In the statistical analysis as well as in the subsequent interpretation of results, the chi-square and *I*-square parameters may not be sufficiently informative as they ignore the threshold effect. As this study follows a random effects model (due to the presence of between-study heterogeneity), the tau-squared statistical parameter, being the estimated variation of heterogeneity between the effects for test accuracy observed in different studies, might be suitable for inclusion in this meta-analysis study. Furthermore, the authors have also stated that Deeks et al.'s test performed showed a statistically nonsignificant value. It is important to consider that Deeks et al.'s test is used to identify the effective same sample size, and it is insufficient in clarifying the presence of patient-level or study-level bias that exists [6].

This study would be a valuable resource for future studies in this field if these issues were to be addressed.
